# Intriguing type-II g-GeC/AlN bilayer heterostructure for photocatalytic water decomposition and hydrogen production

**DOI:** 10.1038/s41598-023-45744-6

**Published:** 2023-10-31

**Authors:** Naim Ferdous, Md. Sherajul Islam, Md. Shahabul Alam, Md. Yasir Zamil, Jeshurun Biney, Sareh Vatani, Jeongwon Park

**Affiliations:** 1 Department of Electrical and Biomedical Engineering, University of Nevada, Reno, NV 89557 USA; 2 Department of Electrical and Electronic Engineering, Khulna University of Engineering and Technology, Khulna, 9203 Bangladesh; 3 School of Electrical Engineering and Computer Science, University of Ottawa, Ottawa, ON K1N6N5 Canada

**Keywords:** Nanoscale materials, Nanoscience and technology, Materials science, Condensed-matter physics, Materials for energy and catalysis, Nanoscale materials, Theory and computation

## Abstract

Adapting two-dimensional (2D) van der Walls bilayer heterostructure is an efficient technique for realizing fascinating properties and playing a key role in solar energy-driven water decomposition schemes. By means of first-principles calculations, this study reveals the intriguing potential of a novel 2D van der Walls hetero-bilayer consisting of GeC and AlN layer in the photocatalytic water splitting method to generate hydrogen. The GeC/AlN heterostructure has an appropriate band gap of 2.05 eV, wherein the band edges are in proper energetic positions to provoke the water redox reaction to generate hydrogen and oxygen. The type-II band alignment of the bilayer facilitates the real-space spontaneous separation of the photogenerated electrons and holes in the different layers, improving the photocatalytic activity significantly. Analysis of the electrostatic potential and the charge density difference unravels the build-up of an inherent electric field at the interface, preventing electron–hole recombination. The ample absorption spectrum of the bilayer from the ultra-violet to the near-infrared region, reaching up to 8.71 × 10^5^/cm, combined with the resiliency to the biaxial strain, points out the excellent photocatalytic performance of the bilayer heterostructure. On top of rendering useful information on the key features of the GeC/AlN hetero-bilayer, the study offers informative details on the experimental design of the van der Walls bilayer heterostructure for solar-to-hydrogen conversion applications.

## Introduction

The development of clean and renewable energy alternatives to fossil fuels has become crucial due to the growing energy and environmental challenges. The superior benefits of hydrogen energy in the form of ultra-cleanness, environmental friendliness, and power make it a potential candidate for energy replacement^1–4^. Nevertheless, the conventional approaches of hydrogen generation, such as biomass pyrolysis, high pressure and temperature steam methane reforming, and gasification of coal (~ 5 MPa), involve CO_2_ emission and are intrinsically expensive^5,6^. In this regard, hydrogen generation through a solar energy-driven water splitting scheme is regarded as an environment-friendly and renewable technique^3,7,8^. Photons from sunlight illuminate a semiconductor, generating electrons and holes. The excited electrons then participate in the water reduction reaction, producing hydrogen, while the excited holes produce oxygen through the oxidation reaction. Nonetheless, developing an effective water-decomposition photocatalysis scheme requires materials with a proper band gap, excellent separation of the carriers for effective surface activity, adequate redox potentials to provoke the redox reaction evenly, along with photochemical steadiness, eco-friendliness, and commercial viability^7^. This indicates that the design of an efficient photocatalyst plays a crucial role in achieving high-efficiency water decomposition.

Following the successful exfoliation of graphene from graphite^9^, two-dimensional materials have received tremendous attention in various fields^10–12^. Recent studies have demonstrated the potential of graphene-like two-dimensional materials, including transition metal dichalcogenides^13,14^, g-C_3_N_4_^15^, transition metal oxides^16^, phosphorene^17^, and MXenes^18^ in optoelectronic and photocatalytic applications. In contrast to bulk semiconductors, two-dimensional materials are ultra-thin and have a highly precise surface area, allowing additional exposed active sites to be involved in the photocatalytic reaction, resulting in better photocatalytic performance^19,20^. Despite this, single 2D materials are still limited in practical photocatalytic application owing to the ineligible electron–hole pair recombination, as well as the incompatibility between large light response ranges (require a narrower band-gap) and strong redox capability (require a greater band-gap)^21,22^. Due to this, different strategies have been investigated to enhance the photocatalytic performance of two-dimensional photocatalytic materials, such as adsorption^23^, defects^24,25^, introducing doping^24,26^, and adapting 2D/2D heterostructures^27–33^. One of these, the method of developing van der Walls (vdW) heterostructures, has the greatest potential due to its capability to separate electron–hole pairs and combine the individual benefits of each constituent layer^34–37^. Generally, type-II heterostructure forms when the corresponding band edges of two constituent semiconductor layers develop a staggered band alignment, where the valence band maximum (VBM) and conduction band minimum (CBM) are occupied by two different layers. This provides a real-space separation of the photo-excited electrons and holes; hindering the recombination of the electron–hole pair significantly and enhancing the photocatalytic activity^38^.

Furthermore, the 2D/2D vdW heterostructures can modify their band structure to meet the prospective needs of photocatalytic reaction^39^. The electron–electron correlations and exciton binding energies are also enriched in these heterostructures through the quantum confinement effect. Besides, due to the intrinsic electric field at their interface, most of the photoexcited carriers are separated into two different layers, extending the lifetime of the carriers. These heterostructures also offer a large contact surface between water and photo-induced carriers, decreasing the migration gap of carriers and inhibiting the electron–hole recombination^39,40^. Moreover, the intrinsic dipole moment of the heterostructure increases the dynamic over-potential of the water oxidation and reduction reaction, improving the overall photocatalytic performance substantially^41^.

Numerous two-dimensional materials have been explored to develop 2D/2D heterostructure photocatalysts, both theoretically^42–47^ and experimentally^30,48^. Graphene-like germanium carbide (g-GeC) based heterostructures are appealing and intriguing candidates for efficient solar-to-hydrogen conversion reaction^28,41,49^. This can be attributed to the wide band gap of g-GeC and its high Poisson’s ratio and low rigidity, which have made it favorable in electronics, optoelectronics, photovoltaics, and heterostructures^50–53^. External aspects, including strain^54^, electric field^55^, and surface functionalization^56^, can engineer the electrical and optical properties of the g-GeC. Besides, monolayer aluminum nitride (AlN), a novel and standard group-III nitride, has drawn immense attention in spintronic, optical, optoelectronic, and substrate application^57,58^. The AlN layer demonstrates comparable symmetry to the GeC layer along with minor lattice mismatch, implying the easy experimental fabrication of the GeC/AlN vdW heterostructure. Wu et al. reported the synthesis of GeC thin films using plasma enhanced chemical vapor deposition (PECVD) technique^59^. Single crystalline AlN thin films are also grown on Si(100) substrate using a helicon sputtering system^60^. However, the vdW heterostructure comprising of g-GeC and AlN monolayer has not yet been studied in literature to analyze its solar to hydrogen conversion performance. Hence, it is significant to develop the GeC/AlN vdW heterostructure and divulge its potential as a photocatalyst for water splitting.

This work demonstrates the GeC/AlN vdW heterostructure as a promising candidate for solar-driven water splitting by employing the first principles calculations. The heterostructure possesses a type-II band alignment wherein the photo-induced carriers are spatially separated in the different layers. The proper energetic positions of the VBM and CBM of the heterostructure provide sufficient kinetic overpotential to drive the water redox reactions. A significant amount of charge is transferred at the interface, building an electric field prohibiting electron–hole recombination. Importantly, ample optical absorption spectrum was found in the visible and near-infrared regions. The effect of incorporating biaxial strain on the photocatalytic performance of the GeC/AlN hetero-bilayer is also studied. The collective outcome of these features indicates the superior performance of the GeC/AlN vdW heterostructure as a photocatalyst to generate hydrogen and shows a new avenue for designing vdW heterostructure based on GeC/AlN for an efficient photocatalysis scheme.

## Calculation methods

First Principles Density Functional Theory (DFT) calculations are applied with the ab-initio total-energy and molecular dynamics package VASP (Vienna ab-initio simulation package) as integrated into the MedeA software environment^61,62^. In order to consider the exchange–correlation interaction among the electrons, we implemented the Perdew-Burke-Ernzerhof scheme within the generalized gradient approximation (PBE-GGA)^63^. The core electrons have been characterized by the projector-augmented-wave (PAW) potentials^64^. As the PBE-GGA functional underestimates the significant van der Walls (vdW) interactions, Grimme’s DFT-D2 correction method^65^ for the vdW interactions (D represents dispersion) has been employed to consider the effect of the non-trivial vdW force on the GeC and AlN layers in the bilayer structure. The kinetic energy cut-off was taken as 500 eV for the plane wave to expand. A 24 × 24 × 1 Monkhorst–Pack (MP) k-point mesh was adopted for geometry optimization and electronic band structure calculation while a 36 × 36 × 1 MP k-grid was utilized for density of states and optical property calculation^66^. The convergence criterion for force was taken as 0.02 eV/Å while for energy the converge criterion was 10^–5^ eV. A vacuum region exceeding 15 Å along the Z-axis was employed to eliminate the interaction between the two successive layers.

Moreover, density functional perturbation theory (DFPT) is employed to calculate the phonon dispersions^67^. Frequency-dependent complex dielectric formula: $\varepsilon \left(\omega \right)={\varepsilon }_{1}\left(\omega \right)+i{\varepsilon }_{2}\left(\omega \right)$ is utilized to model the optical properties. There is a distinct relationship between the electronic dispersion's cross-band energies and the imaginary component of the dielectric function. One can visualize this correlation through the following equation representing the filled and unfilled states:

1 $${\varepsilon }_{2}=\frac{2{e}^{2}\pi }{\Omega {\varepsilon }_{ o}}{\sum }_{k,v,c}{\left|\langle {\psi }_{k}^{c}\left|\widehat{u}\times r\right|{\psi }_{k}^{v}\rangle \right|}^{2}\delta \left({E}_{k}^{c}-{E}_{k}^{v}-E\right)$$

Here, the polarization vector of the occurrence field is represented by $\widehat{u}$, $e$ refers to the charged electron, $r$ stands for the spatial position, ${\psi }_{k}^{c}$ (${\psi }_{k}^{v}$) is the conduction (valence) band wave function at wave vector $k$, polarization density is denoted by $\Omega$ while ${E}_{k}^{v}$, ${E}_{k}^{c}$, and $E$ are the conduction band energy, valence band energy, and the Fermi energy, respectively.

## Results and discussion

**Figure 1:**
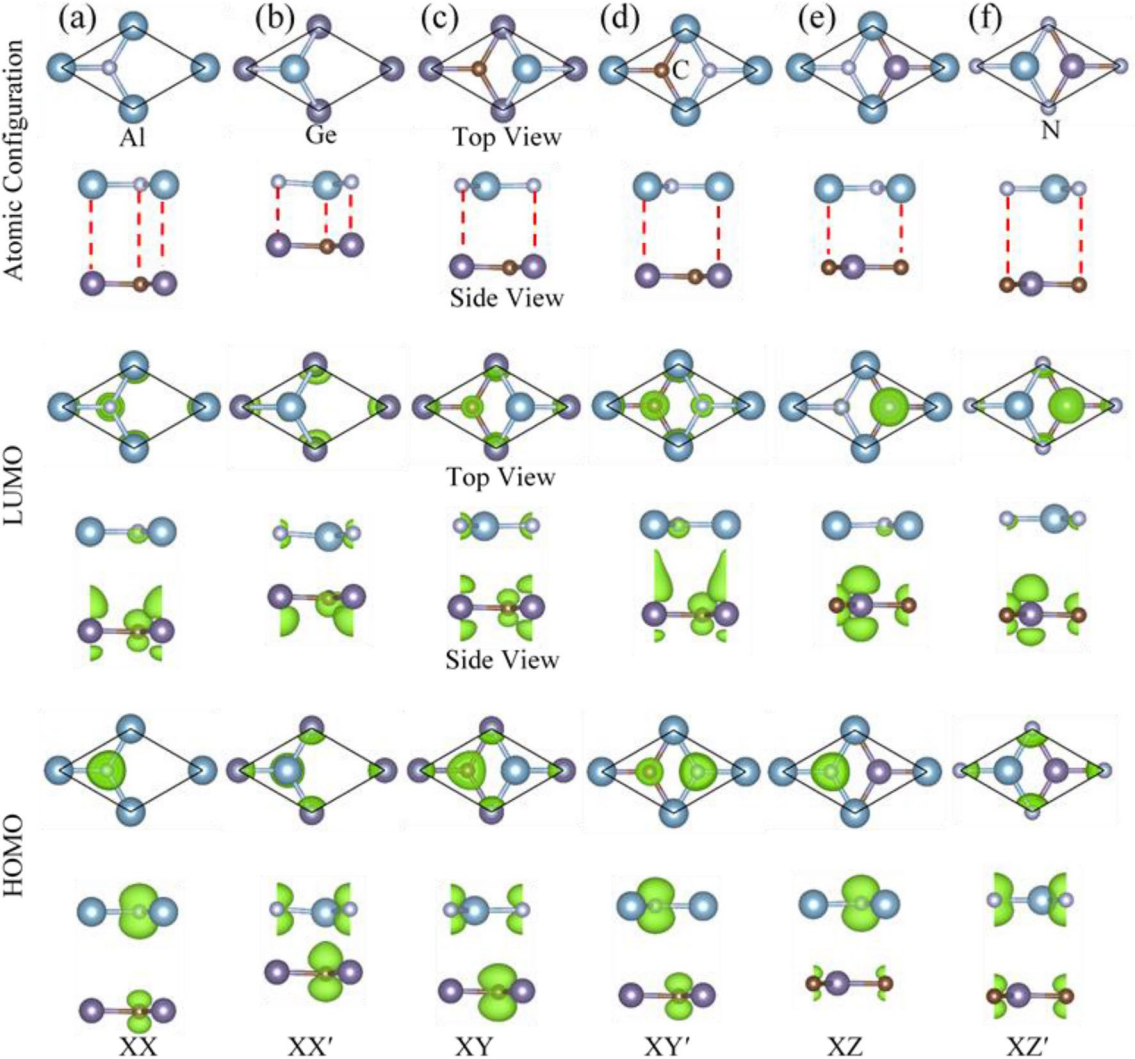
(First row) atomistic model of the GeC/AlN bilayer heterostructure and their band decomposed charge density of the (second row) lowest unoccupied molecular orbital (LUMO) and (third row) highest occupied molecular orbital (HOMO), respectively. Six different configurations are represented by (**a**) XX, (**b**) XX′, (**c**) XY, (**d**) XY′, (**e**) XZ, and (**f**) XZ′.

The GeC/AlN vdW bilayer heterostructure is constructed by vertically stacking monolayer AlN on top of the GeC monolayer. The structural and electronic properties of the freestanding GeC layer and AlN sheet are detailed in the supporting information. The lattice mismatch between the AlN layer and GeC monolayer is 4.07%, which is obtained by $\left|\frac{({a}_{AlN}-{a}_{GeC}}{\left({a}_{AlN}+{a}_{GeC}\right)/2}\right|$, where ${a}_{GeC}$ and ${a}_{AlN}$ are the geometry optimized lattice constants of the GeC and AlN monolayers, respectively. The minimal lattice mismatch between the two different layers implies the feasibility of achieving the intended hetero-bilayer by vertically stacking the 2D-AlN and GeC monolayer. As Hu et al. report^68^, it is rewarding if a heterostructure is constructed with a lattice mismatch not exceeding 5%. In contrast to the bilayer graphene, GeC/AlN hetero-bilayer can be stacked in six different configurations because the atoms in each layer are different. The six geometry-optimized stacking configurations of the GeC/AlN bilayer heterostructure are illustrated in Fig. 1 and are denoted as XX, XX′, XY, XY′, XZ and XZ′. The XX configuration is attained by placing the N atom on top of the C atom and the Al atom on top of the Ge atom. In the XY stacking pattern, the N atom is on top of the Ge atom, whereas the Al and C atoms are on the hexagonal side. The XZ pattern is obtained by locating the Al atom directly above the C atom while the Ge and N atoms are on the hexagonal side. The XX′, XY′, and XZ′ stacking configurations are the inverse of XX, XY, and XZ configurations, respectively. The band decomposed charge densities of the highest occupied molecular orbital (HOMO) and the lowest unoccupied molecular orbital (LUMO) of the six configurations are also included in Fig. 1. As Fig. 1 suggests, for the XZ configuration, the HOMO is mainly contributed by the N atom of the AlN layer, while the LUMO comes mostly from the Ge and C atoms of the GeC layer. The localization of the HOMO and the LUMO in two different layers indicates that the XZ configuration of the GeC/AlN bilayer has a type-II heterojunction.

The GeC/AlN bilayer heterostructure is comparable to the 2D boron phosphide/SiC vdW heterostructure studied by Do et al.^69^. Due to a very small lattice mismatch of 1.75% between boron phosphide and layered SiC, the heterostructure was formed by stacking the boron phosphide layer on top of the SiC layer, and six similar stacking configurations were considered to reveal its potential as high-efficiency photo-catalyst. Lou and Lee, while analyzing GeC/GaN vdW heterojunctions for photocatalytic water splitting, also constructed six possible patterns of the heterojunction owing to 0.63% lattice mismatch between the GeC and GaN layer^41^. In addition, in the 2D NbSe_2_H/g-ZnO vdW heterostructure^70^, the lattice mismatch was 4.59%. Therefore, six high-symmetry stackings were adopted for its theoretical prediction as a water-splitting photocatalyst.

After geometry-relaxation, the lattice constants of the six stacking patterns XX, XX′, XY, XY′, XZ, and XZ′ are 3.198 Å, 3.255 Å, 3.203 Å, 3.199 Å, 3.205 Å, and 3.198 Å, respectively (listed in Table S1). The structural stability of a bilayer heterostructure can be evaluated by calculating its binding energy. A negative binding energy value suggests that the structure is energetically stable. On the other hand, positive binding energy indicates structural instability of the system. The following formula has been utilized to obtain the binding energy of the GeC/AlN vdW bilayer heterostructure:

2 $${E}_{B}=\frac{{E}_{GeC/AlN}-{E}_{GeC}-{E}_{AlN}}{A}$$

where ${E}_{B}$ refers to the binding energy of the GeC/AlN vdW bilayer heterostructure, and A is the heterostructure’s interface area. ${E}_{GeC/AlN}$, ${E}_{GeC}$, and ${E}_{AlN}$ stand for the total energy of the GeC/AlN vdW heterobilayer, free-standing GeC monolayer and free-standing AlN layer, respectively. Table S1 enrolls the binding energies of all the structural configurations. Obtained binding energy values of the six patterns are negative, suggesting that they are energetically favorable. Besides, the binding energies of the six patterns range from − 2.47 to − 45.16 meV/Å^2^, indicating that weak vdW forces dominate between the two layers in the bilayer structure. Figure S2 illustrates the change of the binding energy with respect to the inter-layer distance for the six structures. The optimized interlayer distances (h_o_) of the six stacking patterns are enlisted in Table S1 and extend from 2.43 to 3.82 Å. The values of h_o_ are comparable to the interlayer spacing of regular vdW heterostructure, for example, graphite. In this regard, while studying ZnO/GaN vdW bilayer structure as photocatalyst^71^, Ren et al. also obtained similar values of interface distance (2.41–3.69 Å) for the heterostructure. Besides, the optimized interlayer distances of the GeC/AlN bilayer structure are higher than the covalent bond length of the Al–C bond (2.01 Å) and the Ge–N bond (1.92 Å). This suggests the absence of covalent bonding between the GeC and AlN layers in the heterostructure.

**Figure 2:**
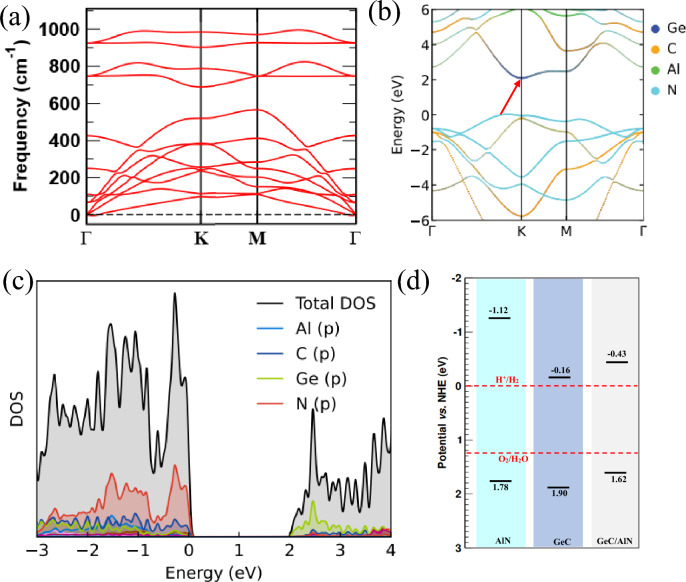
(**a**) Phonon dispersion relations of the XZ configuration of the g-GeC/AlN bilyaer heterostructure. Atom projected (**b**) band structure and (**c**) density of states of the GeC/AlN heterostructure calculated employing the PBE-GGA functional. (**d**) The VBM and CBM energy locations of the pristine AlN, GeC layer and the GeC/AlN bilayer heterostructure with respect to normal hydrogen electrode (NHE) potential. The red dotted lines refer to the oxygen evolution and hydrogen evolution reaction potential with respect to NHE.

Next, the electronic properties of the GeC/AlN bilayer heterostructure are investigated. Figure S3 represents the electronic band structure of the six patterns of the heterostructure, while the electronic band gaps and the VBM and the CBM positions of the six structures are summarized in Table S1. XX, XY′, and XZ′ configurations have direct band gaps, while the three other configurations show indirect band gaps. The XY′ configuration has the lowest band gap (1.793 eV), while it is the maximum for the XX′ configuration (2.834 eV). From the atom-projected band structures of the six stacking configurations (Fig. S3), one can observe that the XZ stacking configuration has a type-II (staggered) band alignment wherein the VBM and CBM are localized from two different layers. Such a type-II alignment is highly efficient for solar energy conversion owing to the spontaneous separation of electrons and holes. Therefore, for our further study on the GeC/AlN bilayer heterostructure to reveal its intriguing potential as an efficient photocatalyst for water decomposition, we focus on the XZ configuration of the GeC/AlN hetero-structure. The dynamic stability of the GeC/AlN vdW hetero-bilayer is assessed by calculating its phonon spectrum. The phonon spectrum of the XZ configuration of the GeC/AlN hetero-bilayer is portrayed in Fig. 2a. The absence of soft frequency in all the phonon branches through the whole Brillouin zone indicates the dynamic stability of the GeC/AlN vdW bilayer. Next, the atom projected band structure of the GeC/AlN vdW heterostructure (XZ configuration) is shown in Fig. 2b, where the navy-blue, cyber-yellow, green, and sky-blue colors refer to the contribution from Ge, C, Al, and N atom, respectively. As the red arrow indicates from VBM to CBM, one can clearly observe that VBM is contributed by the N atom while the CBM originates from the Ge atom. Thus, the GeC/AlN hetero-bilayer demonstrates a type-II band alignment where the CBM and VBM are localized from the GeC and AlN layers, respectively. Figure 2c illustrates the corresponding total density of states (TDOS) and projected density of states (PDOS) of the GeC/AlN heterostructure. It is apparent that, around the VBM, the dominating contribution comes from the N-p state of the AlN monolayer while the CBM is occupied by the Ge-p state of the GeC monolayer, further confirming the typical type-II band orientation in the GeC/AlN hetero-bilayer. The type-II band orientation facilitates the spatial separation of the photogenerated electrons and holes effectively through the accumulation of electrons and holes in two distinct monolayers. The GeC/AlN bilayer, thus, appears to be an intriguing candidate for photocatalytic water decomposition and photovoltaic devices wherein the electron–hole-recombination rate is reduced greatly^39,72^.

Proper band edge positions of the semiconductor are basic criteria for efficient solar energy-driven water splitting. Hence, the band edge positions of the valence band and the conduction band of the GeC/AlN vdW bilayer heterostructure are determined with respect to normal hydrogen electrode (NHE) potential. The Mulliken electronegativity equation is used to calculate the band edge location of the heterostructure^73,74^. The valence band edge (${E}_{VB}$) and the conduction band edge (${E}_{CB}$) at the neutral environment (pH = 0) are determined employing the following relations:

3 $${E}_{CB}=\upchi -{E}_{e}-\frac{{E}_{g}}{2}$$

4 $${E}_{VB}={E}_{CB}+{E}_{g}$$

Here, $\chi$ stands for the geometric mean of the constituent atom’s Mulliken electronegativity, and ${E}_{g}$ is the obtained electronic band gap value of the material. ${E}_{e}$ represents the free electron’s energy in hydrogen scale with a value of 4.5 eV. The value of $\chi$ for the GeC/AlN vdW bilayer is 5.09 eV. A semiconductor needs to satisfy certain criteria to produce hydrogen and oxygen by photocatalytic water decomposition scheme. The valence band edge of the semiconductor needs to be equal to or greater than 1.23 eV with respect to NHE potential so that the photogenic holes trigger the water oxidation reaction (O_2_/H_2_O) at a neutral environment while the CBM energy level is necessary to be equal or less than 0 eV in regard to NHE potential to initiate the reduction reaction (H+/H_2_) by the photogenerated electrons. Therefore, the semiconducting material should have a minimum band gap of 1.23 eV, which needs to straddle the water oxidation and reduction potential. Moreover, the material should exhibit significant absorption peaks in the near ultraviolet and visible region to utilize solar energy effectively. What’s more, the semiconductor needs a higher surface-to-volume ratio to promote the redox reaction to split the water.

The VBM and CBM energy levels of the GeC/AlN bilayer heterostructure, along with those of the freestanding AlN and GeC layer, are plotted in Fig. 2d. The red dotted lines represent the water oxidation reaction (oxygen evolution reaction) and reduction reaction (hydrogen evolution reaction) potentials with respect to NHE in the neutral environment. As our calculation yields, the energy gap of the pristine AlN layer spans from 1.78 to − 1.12 eV, straddling the O_2_/H_2_O and H+/H_2_ reaction potential. In the case of a freestanding GeC monolayer, the VBM and CBM energy levels are 1.90 eV and − 0.16 eV, which are 0.67 eV and 0.16 eV higher and lower than the oxygen evolution reaction and hydrogen evolution reaction potential, respectively. The electronic band gap of the GeC/AlN bilayer is extending from 1.63 to − 0.42 eV, which is also 0.4 eV higher and 0.42 eV lower than the O_2_/H_2_O and H^+^/H_2_ reaction potential in regard to NHE, respectively. The AlN sheet, the GeC monolayer, and the GeC/AlN bilayer all possess adequate kinetic over-potential to promote the photocatalytic water decomposition scheme. However, freestanding AlN and GeC monolayers do not possess type-II band alignment to separate the photogenerated electrons and holes spatially. The GeC/AlN bilayer structure, therefore, appears to be an excellent candidate for a solar energy-driven water splitting scheme, facilitating the real space separation of the electrons and holes in its distinct layers with the type-II band configuration and inducing the O_2_/H_2_O and H^+^/H_2_ reactions to generate hydrogen.

The Heyd-Scuseria-Ernzerhof (HSE06) hybrid functional^75^ is well-known to produce electronic band gap close to experiments. Therefore, the HSE06 functional was also adopted for a more precise calculation of the electronic structure. Figure S4a illustrates the electronic band structure of the GeC/AlN bilayer heterostructure obtained by the HSE06 functional. The HSE06 functional yields an electronic band gap of 2.94 eV for the GeC/AlN vdW heterostructure. Besides, the VBM and CBM energy positions of the heterostructure are also calculated employing Eqs. (3) and (4) with this band gap. The band edge positions of the hetero-bilayer are depicted in Fig. S4b. After adopting the HSE06 functional, the band gap extends from 2.06 to − 0.88 eV; which straddles the O_2_/H2O and H^+^/H_2_ reaction potential. Thus, the HSE06 adopted electronic band structure demonstrates sufficient kinetic overpotential to trigger the hydrogen evolution and oxygen evolution reactions.

**Figure 3:**
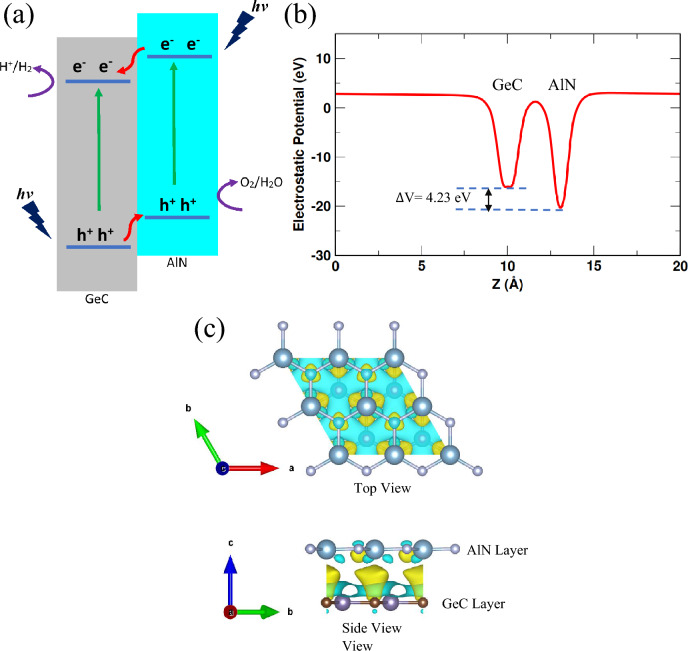
(**a**) Schematic representation of the charge transfer in type-II GeC/AlN vdW heterostructure under solar irradiation. (**b**) Plane-averaged electrostatic potential of the GeC/AlN bilayer for the XZ pattern. ∆V refers to the potential drop across the interface. (**c**) Three-dimensional (3D) iso-surface of the charge density difference of the heterostructure with an iso-value of 0.0005 e/Å^3^. The yellow and cyan color correspond to the increase and decrease of charge, respectively.

Figure 3a describes the photocatalytic water division mechanism to generate hydrogen and oxygen in the GeC/AlN bilayer structure as well as the transfer of photogenic electrons and holes in type-II band configuration. After solar radiation illuminates the GeC/AlN semiconductor and the energy of the photons exceeds the electronic band gap of the AlN and GeC layers, photogenic electrons will begin moving to the conduction band of the GeC and AlN layer from the valence band, resulting in the formation of holes in the valence band of the AlN and GeC layers. When the electrons get to the valence band of AlN, the valence band offset ($\Delta {E}_{C}$) promotes the electrons to migrate to the valence band of GeC. In contrast, conduction band offset ($\Delta {E}_{V}$) promotes the holes to migrate from the GeC layer’s valence band to the AlN layer's valence band. The $\Delta {E}_{C}$ and $\Delta {E}_{V}$ are calculated as 0.96 eV and 0.12 eV, respectively. Thus, the photogenic electrons and holes are spatially separated in the GeC layer and the AlN layer contributing to the H^+^/H_2_ and O_2_/H_2_O reactions, respectively. Hydrogen production will occur at the GeC sheet, while oxygen will be generated in the AlN sheet.

The plane-averaged electrostatic potential of the GeC/AlN bilayer heterostructure is plotted in Fig. 3b. One can clearly observe that the AlN layer has more profound electrostatic potential than the GeC layer. Therefore, negative charges accumulate on the GeC layer while positive charges on the AlN layer. This results in a built-in electric field pointing from the AlN layer to the GeC layer. The potential drop ($\Delta$V) across the heterobilayer is 4.23 eV, as shown in Fig. 3b. The built-in electric field drives the electrons and the holes to move in two different directions, increasing the lifetime of the photogenerated carriers, which further prohibits the electrons and holes to recombine^76^. Consequently, the photocatalytic performance of the GeC/AlN vdW heterostructure is significantly improved. To shed light on the improved photocatalytic activity of the GeC/AlN bilayer, the three-dimensional charge density difference of the heterostructure is calculated. The following formula is utilized to obtain the three-dimensional charge density difference ($\Delta \rho$) of the bilayer:

5 $$\Delta \rho ={\rho }_{GeC/AlN}{-\rho }_{GeC}{-\rho }_{AlN}$$

Here, ${\rho }_{GeC/AlN}$, ${\rho }_{GeC}$, and ${\rho }_{AlN}$ refer to the charge density of the GeC/AlN heterobilayer, GeC monolayer, and AlN monolayer, respectively. Figure 3c represents the three-dimensional charge density difference of the bilayer heterostructure. The yellow color corresponds to the charge increase while the cyan color indicates the decrease of the charge. An apparent change in charge density at the heterostructure interface can be noticed, attributable to the interaction between the GeC layer and the AlN layer. As Fig. 3c indicates, charge re-distribution mostly occurs at the heterostructure interface region. Charges are accumulated in the interface area adjacent to the C atom while the charges are depleted mainly from the interface boundaries. Thus, a polarized field is generated, specifically near the interface, which hinders the photogenic carriers from recombining and promotes the photocatalytic activity^42^. Besides, Bader charge analysis is also employed to determine the total amount of charges transferred at the GeC-AlN interface. It shows that a charge transfer of 0.0215e occurs from GeC to the AlN layer. In addition, owing to the charge transfer at the heterostructure interface and built-in electric filed, electronic band structure and relevant band edge locations are calculated considering the dipole correction which are provided in the supporting information.

**Figure 4:**
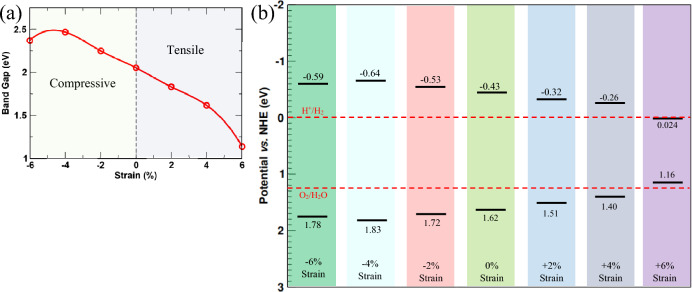
(**a**) Electronic band gap obtained from PBE-GGA functional and (**b**) evolution of the VBM and CBM energetic positions (with respect to normal hydrogen electrode) as a function of percentage biaxial strain applied to the GeC/AlN heterostructure in the range of − 6% to + 6%.

Interestingly, a semiconductor's electrical, optical, and transport behavior can be mediated by applying strain on it^77^. It is especially useful for engineering 1D and 2D crystals since these reduced-dimensional structures can sustain greater strains in contrast to the bulk crystals^78–80^. Therefore, we studied the effect of biaxial strain on the electrical and optical properties of the GeC/AlN bilayer structure. The lattice constant of the bilayer structure after applying biaxial strain on it ($a$) is defined by the following relation:

6 $$a={a}_{0}(1+\varepsilon )$$

where ${a}_{0}$ is the relaxed lattice constant of the GeC/AlN bilayer heterostructure and $\varepsilon$ refers to the biaxial strain applied onto the structure. Positive value of $\varepsilon$ corresponds to the tensile strain while its negative value indicates compressive strain. The bilayer was subject to the biaxial strain extending from − 6 to + 6% with an increase of 2%. Figure S6 represents the electronic band diagram of the heterostructure under varied percentage of strain. The changing trend of the band-gap value with the change of the biaxial strain applied to the structure is demonstrated in Fig. 4a. As Fig. 4a suggests, compressive strain causes the band-gap value to increase while it is decreased through applying tensile biaxial strain. The band edge positions of the GeC/AlN bilayer heterostructure under the varied percentage of biaxial strain is shown in Fig. 4b. The bilayer structure’s VBM and CMB energy positions are in suitable locations to induce the hydrogen evolution reaction and the oxygen revolution under strain ranging from − 6 to + 4%. Within this range, with the increase of strain, although the band edges are getting closer to the water oxidation and reduction potential, the VBM and CBM energy locations are still in a favorable position to induce the water redox reaction. But, at + 6% strain, the CBM energy position is below the H^+^/H_2_ potential to initialize the reduction reaction. However, within − 6% to + 4% strain, proper band edges of the heterostructure to induce the water redox reaction imply the bilayer’s resiliency to the strain for the photocatalytic water decomposition scheme. Moreover, the dynamic stability of the heterostructure under strain is assessed by obtaining the phonon dispersion relations of the structure under varying percentage of biaxial strain. Figure S7 portrays the phonon spectrum of the bilayer structure under strain from − 6 to + 6%. As the figure suggests, for tensile strain from + 2 to + 6%, there is no imaginary frequency (soft mode) through the entire Brillouin zone; implying that the heterostructure is dynamically stable under tensile strain up to + 6%. However, while the heterostructure is under compressive strain (− 2% to-6%), soft frequencies are present in the Brillouin zone of phonon spectrum. This indicates that the structure may not be dynamically stable under compressive strain.

**Figure 5:**
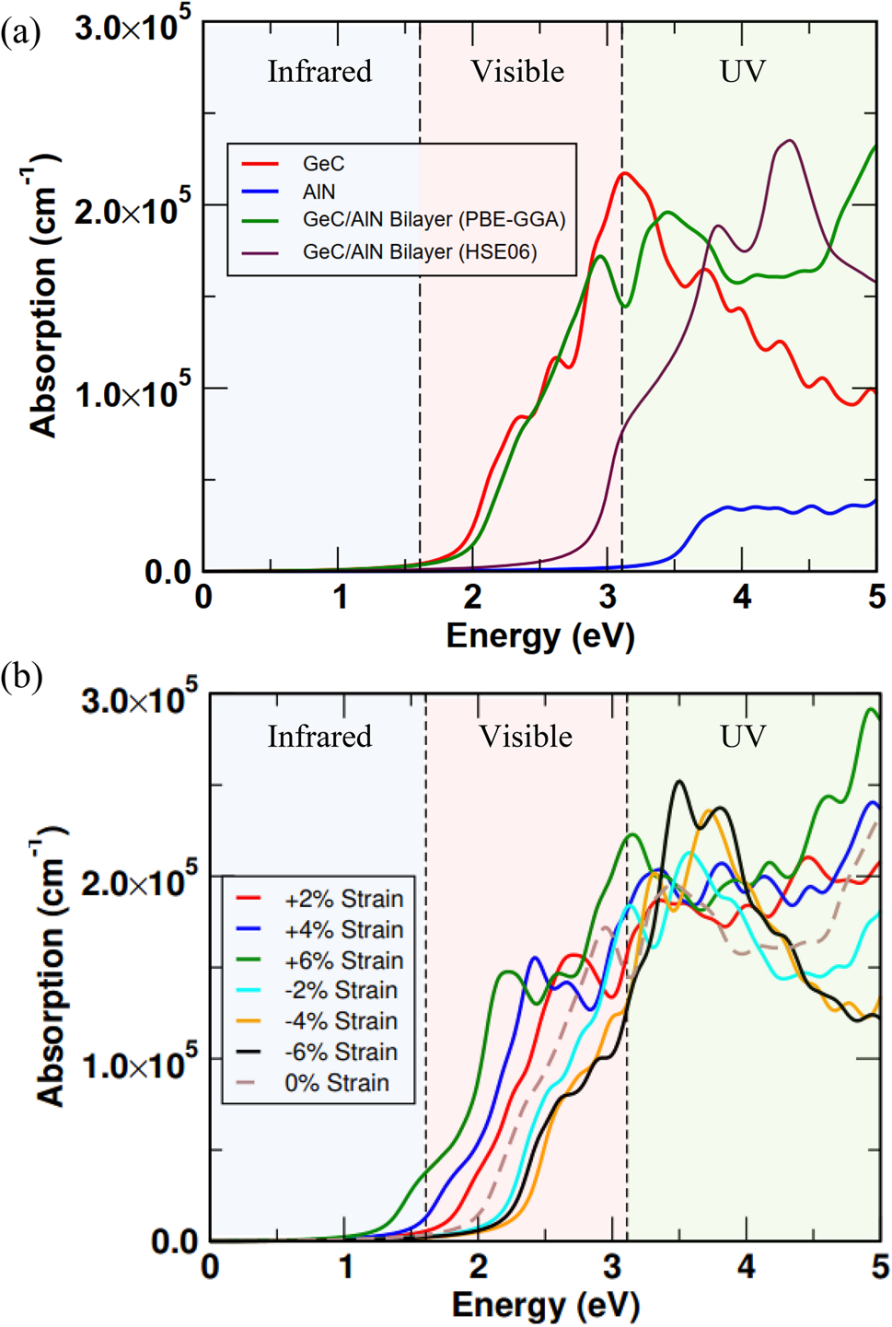
Optical absorption spectra of the (**a**) 2D AlN, 2D GeC and GeC/AlN vdW bilayer heterostructure and (**b**) the GeC/AlN bilayer under varied percentage of biaxial strain from − 6 to + 6%.

A material’s optical properties can be determined through its absorption spectrum. The complex dielectric function $\varepsilon (\omega )$ is calculated and the following relation has been utilized with a view to evaluating the optical absorption coefficient $\alpha (\omega )$:

7 $$\alpha (\omega )=\sqrt{2}\omega \sqrt{\left[\sqrt{{\varepsilon }_{1}^{2}(\omega )+{\varepsilon }_{2}^{2}(\omega )}-{\varepsilon }_{1}(\omega )\right]}$$

where ${\varepsilon }_{1}(\omega )$ is the real part and ${\varepsilon }_{2}(\omega )$ is the imaginary part of the frequency-dependent complex dielectric function. Figure 5a shows the absorption coefficient of the GeC/AlN vdW heterostructure obtained by the PBE-GGA function as well as by the HSE06 functional. The absorption coefficients of the pristine GeC and AlN monolayers are also included for comparison. The heterostructure exhibits a wide optical absorption spectrum extending from ultra-violet to the near-infrared region. In particular, we obtained a high absorption peak of the GeC/AlN bilayer structure in the ultra-violet region, attaining 8.71 × 10^5^/cm (The absorption peak of the bilayer attained by the HSE06 functional is 9.62 × 10^5^/cm). In contrast to the GeC and AlN monolayers, the absorption coefficient of the heterostructure is notably increased in the ultra-violet and visible regions. This is attributable to the lower band gap of the hetero-bilayer in comparison to the constituent monolayers, which causes an enhancement in the optical absorption profile of the bilayer. Consequently, the GeC/AlN vdW heterostructure appears to be an outstanding absorber for solar irradiation, enabling an effective solar-driven water division scheme. When the absorption points in the structure are intense and numerous, the photo-catalysis reaction is enhanced, and electron–hole pairs are produced at a higher rate. In addition, biaxial strain can result in the absorption profile of the material shifting from the ultra-violet to the visible regime, facilitating more efficient solar utilization. Figure 5b illustrates the optical absorption spectra of the GeC/AlN vdW bilayer heterostructure when the structure is subject to biaxial strain ranging from − 6 to + 6%. One can observe that when the bilayer structure is subject to tensile strain, in comparison to the absorption profile of the relaxed structure, its absorption profile is significantly enhanced in the visible regime. The optical absorption coefficient in the visual region is maximum at + 6% biaxial strain. On the other hand, with the increase of the compressive strain, the absorption profile of the hetero-bilayer is shifted from the visible region to the ultraviolet region with the decrease of the absorption coefficient in the visible region. This finding can be attributed to the increase and decrease of the electronic band gap of the heterostructure when compressive and tensile strain is incorporated into it, respectively. Accordingly, biaxial strain is an effective tool to tune the optical performance of the GeC/AlN hetero-bilayer while tensile strain enhances the absorption profile of the bilayer in the visible region.

## Conclusion

To conclude, a systematical study has been carried out to reveal the photocatalytic potential of the GeC/AlN vdW hetero-bilayer in a water splitting scheme using first-principles DFT calculations. The vdW heterostructure has a type-II band alignment wherein the VBM and the CBM come differently from the GeC layer and the AlN layer, respectively, enabling the real space separation of the photogenerated electrons and holes in two different layers. The band edges of the bilayer are adequately energetic to induce the hydrogen evolution reaction and the oxygen revolution reaction. The electrostatic potential and the charge density difference analysis reveal the building-up of an inherent electric field at the bilayer interface, which prohibits the electronic-hole recombination and enhances the photocatalytic performance substantially. Subsequently, hydrogen and oxygen will be generated separately at the GeC layer and the AlN layer, respectively. The bilayer material shows an ample optical absorption profile from the ultraviolet to the near-infrared region. Strain engineering can tune the optical absorption profile of the hetero-bilayer while the tensile strain improves the optical absorbance in the visual region. The combination of these fascinating characteristics demonstrates the tremendous promise of the GeC/AlN vdW bilayer heterostructure in solar-driven water decomposition schemes.

### Supplementary Information

Supplementary Information.

## Data Availability

The data that support the findings of this study are available from the corresponding author upon reasonable request.
