# Supplementary material for: Intriguing type-II g-GeC/AlN bilayer heterostructure for photocatalytic water decomposition and hydrogen production
Source: Sci Rep. 2023 Oct 31;13:18778. doi: 10.1038/s41598-023-45744-6 (PMC10618537; doi:10.1038/s41598-023-45744-6)
Supplement: Supplementary file 1 — Supplementary Information. [file 41598_2023_45744_MOESM1_ESM.docx]

Supporting Information

**Intriguing Type-II g-GeC/AlN Bilayer Heterostructure for Photocatalytic Water Decomposition and Hydrogen Production**

Naim Ferdous^1^, Md. Sherajul Islam^1,2,*^, Md. Shahabul Alam^1^, Md. Yasir Zamil^1^, Jeshurun Biney^1^, Sareh Vatani^1^, Jeongwon Park^1,3^

^1^Department of Electrical & Biomedical Engineering, University of Nevada, Reno, NV 89557, USA.

^2^Department of Electrical & Electronic Engineering, Khulna University of Engineering and Technology, Khulna-9203, Bangladesh.

^3^School of Electrical Engineering and Computer Science, University of Ottawa, Ottawa, ON K1N6N5, Canada.

***Corresponding Author’s Email:** [**sheraj_kuet@eee.kuet.ac.bd**](mailto:sheraj_kuet@eee.kuet.ac.bd) **(Md. Sherajul Islam)**


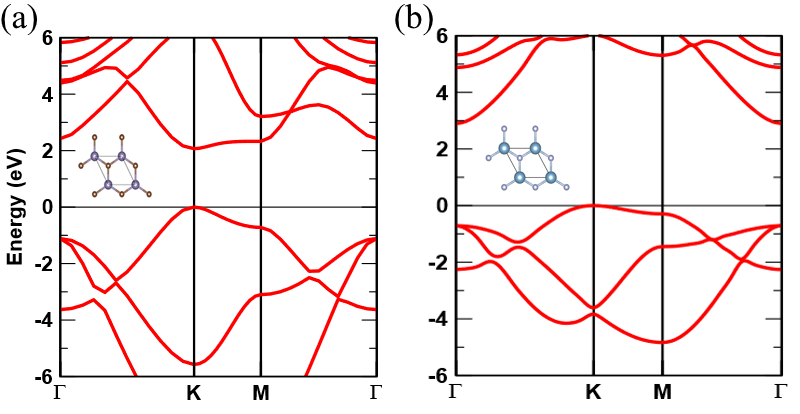


**Figure S1:** Electronic band diagram of (a) GeC monolayer and (b) free standing AlN layer calculated using the PBE-GGA functional. The valence band maximum is set to the zero value of energy. The insets show the top views of the GeC and AlN monolayers.

After geometry optimization, the lattice constant of the freestanding GeC monolayer is calculated as 3.26 Å while the Ge-C bond length is 1.88 Å. The values agree well with the earlier theoretical and experimental studies^1–3^. On the other hand, the geometry-optimized lattice constant and the Al-N bond length of the freestanding AlN monolayer are obtained as 3.13 Å and 1.81 Å, respectively, which are also in close agreement with previous works. The electronic band structures of the pristine GeC layer and monolayer AlN are represented in Figure S1, with their optimized structures shown in the offset. Freestanding layered GeC is a semiconductor with a direct band gap of 2.07 eV. The valence band maximum (VBM) and the conduction band minimum (CBM) are located in the high symmetry K point. The result aligns closely with the work of Ji et al.^2^ (Direct band gap of 2.08 eV) and Yang et al.^4^ (2.07 eV band gap). The VBM of the GeC layer comes mainly from the C-p_z_ orbit, while the CBM is dominated by the Ge-p_z_ orbit^5^. In addition, freestanding monolayer AlN also reveals semiconducting properties with an indirect band gap of 2.91 eV. CBM is located in the $\Gamma$ point, while the VBM is in the K point. This result also corresponds well with the literature^6–8^. N-p_z_ orbital dominates in the VBM of the freestanding AlN layer while the CBM comes from the Al-s and N-s orbitals^7^.


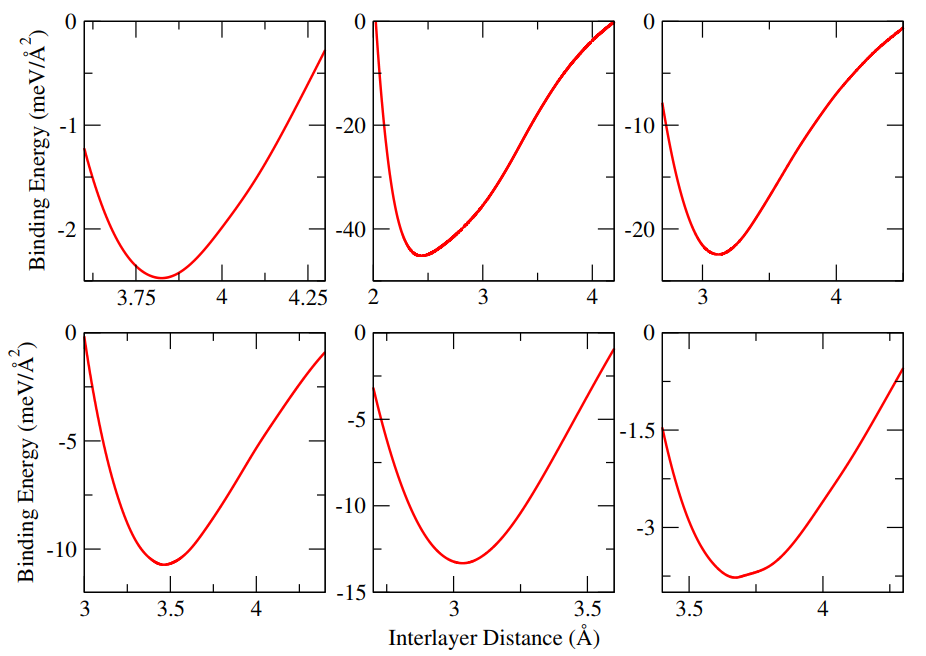


(a)

(b)

(c)

(d)

(e)

(f)

**Figure S2:** Binding energy as a function of interlayer distance for the six structural configurations of the GeC/AlN bilayer heterostructure (a) XX, (b) XX′, (c) XY, (d) XY′, (e) XZ, and (f) XZ′.


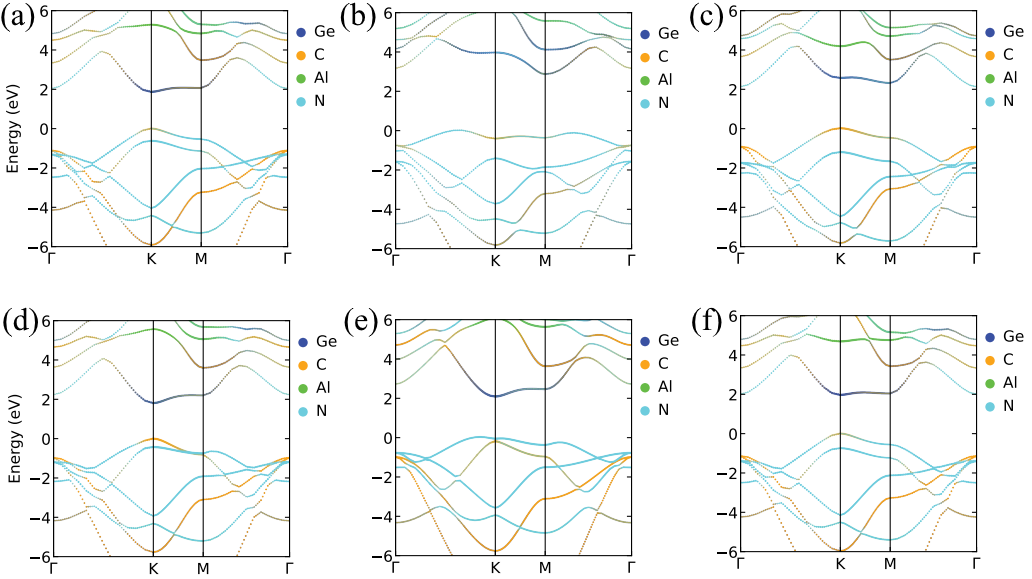


**Figure S3:** Atom projected band structures of the six structural configurations of the GeC/AlN bilayer heterostructure (a) XX, (b) XX′, (c) XY, (d) XY′, (e) XZ, and (f) XZ′ obtained by the PBE-GGA functional. The blue, cyber yellow, green and sky-blue colors refer to the contribution from Ge, C, Al and N atoms, respectively.


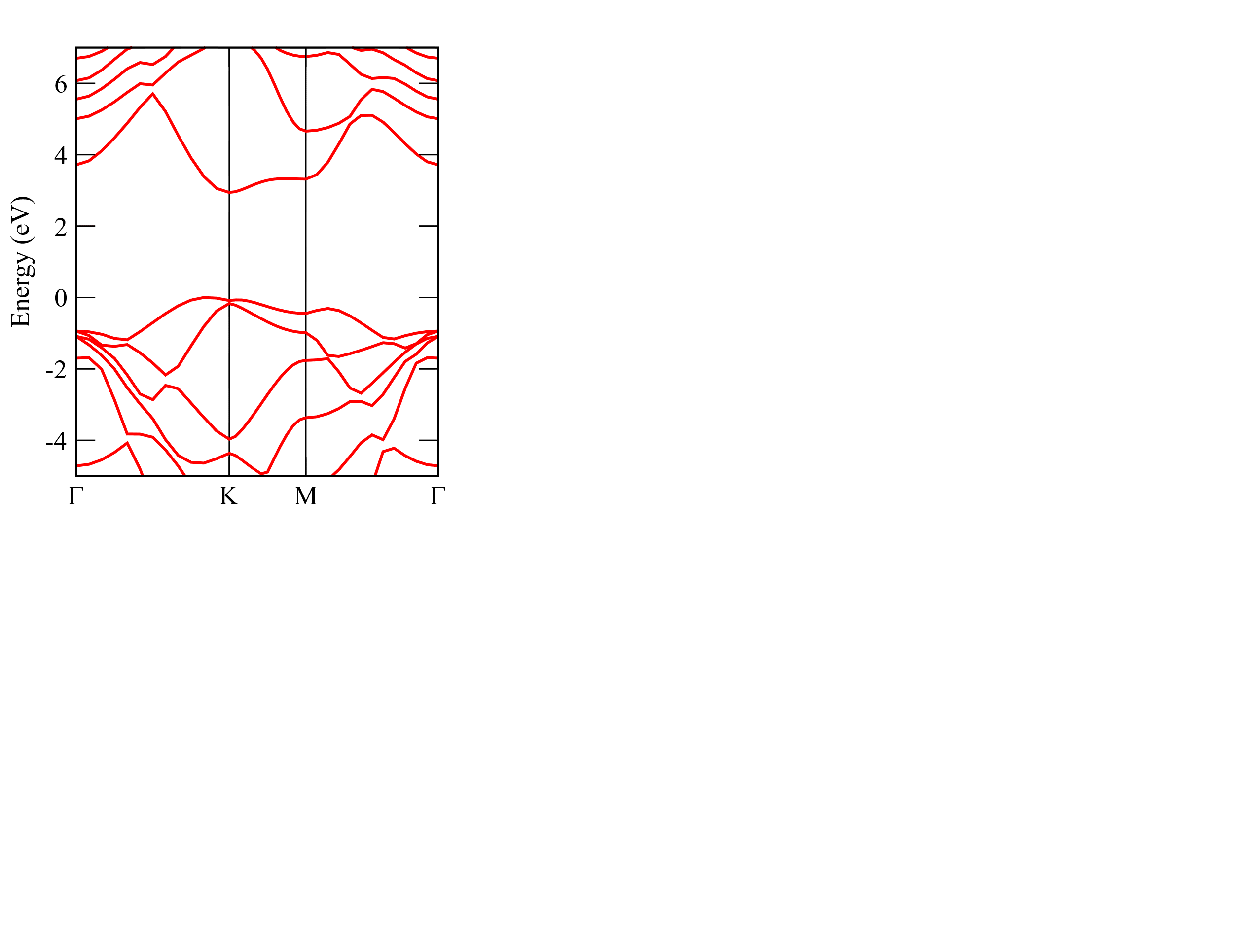


E_g_=2.948 eV

**(a)**

**(b)**

**Figure S4:** (a) Electronic band structure of the g-GeC/AlN hetero-bilayer (XZ configuration) calculated employing the HSE06 hybrid functional. The arrow indicates from VBM to CBM, while the band gap (E_g_) is 2.948 eV. (b) The relevant VBM and CBM energy positions obtained after the HSE06 band structure calculation.

**Figure S5:** (a) Electronic band structure of the g-GeC/AlN bilayer heterostructure considering the dipole correction obtained from PBE-GGA functional. (b) Relevant band edge positions of the heterostructure after the dipole correction.


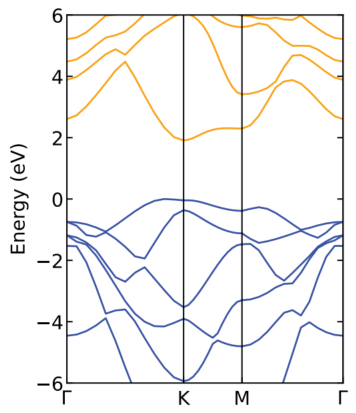


**(a)**

**(b)**

Due to the charge transfer between GeC and AlN layer and built-in electric field in the g-GeC/AlN bilayer heterostructure, the electronic band structure is calculated considering the dipole correction. Figure S5(a) illustrates the band structure of the heterostructure considering the dipole correction. The band gap we obtained after the dipole correction is 1.91 eV; while the band structure resembles the similar shape. The relevant band edge positions are shown in Figure S5(b). The band gap spans 1.545 eV to -0.365 eV. The VBM and CBM energy levels are 0.315 eV and 0.365 eV higher and lower than the oxygen evolution reaction and hydrogen evolution reaction potential, respectively.


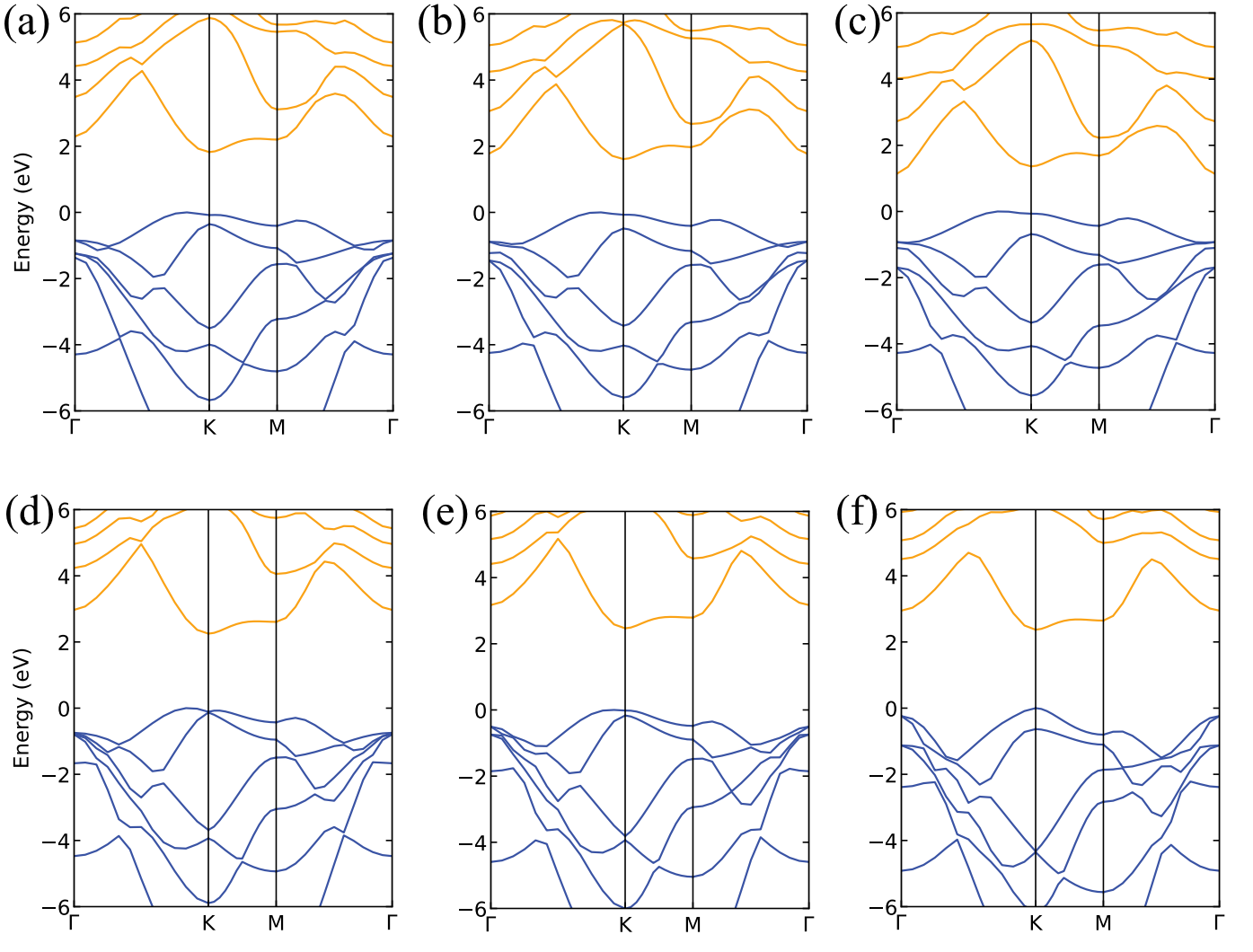


**Figure S6:** Electronic band diagram of the GeC/AlN bilayer heterostructure under varying percentage of strain (a) +2% Strain (b) +4% Strain (c) +6% Strain (d) -2% Strain (e) -4% Strain (f) -6% Strain calculated employing the PBE-GGA functional.


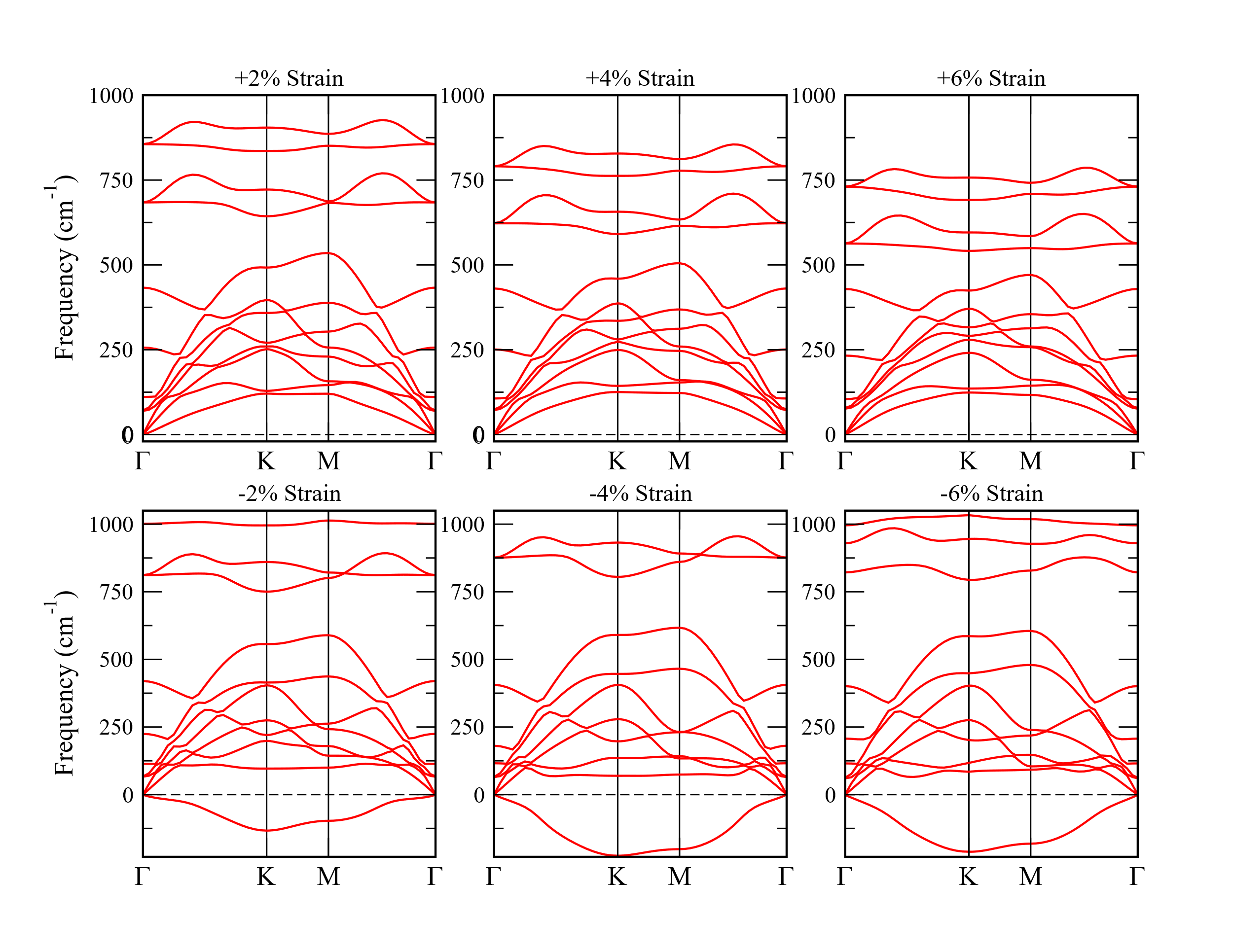


**(a) (b) (c)**

**(d) (e) (f)**

**Figure S7:** Phonon dispersion curves of the g-GeC/AlN bilayer heterostructure under varying percentage of biaxial strain: (a) +2% Strain, (b) +4% Strain, (c) +6% Strain, (d) -2% Strain, (e) -4% Strain, (f) -6% Strain. ‘+’ corresponds to tensile strain while ‘-’ sign corresponds to compressive strain.

**Table S1:** Geometry optimized lattice constant, binding energy per interface area, optimized interlayer distance (h_o_), calculated electronic band gap and the valence band maximum (VBM) and conduction band minimum (CBM) positions of the six configurations of the GeC/AlN vdW heterostructure.

| Stacking Configuration | Optimized lattice Constant ($Å)$ | Binding energy (meV$/Å$^2^) | Optimized interlayer distance ($Å)$ | Band gap (calculated with PBE-GGA in eV) | VBM Position | CBM Position |
| --- | --- | --- | --- | --- | --- | --- |
| XX | 3.198 | -2.47 | 3.82 | 1.853 | K Point | K Point |
| XX′ | 3.255 | -45.16 | 2.43 | 2.834 | $\Gamma-K$ route | $M$point |
| XY | 3.203 | -22.5 | 3.13 | 2.099 | K Point | $\Gamma$ point |
| XY′ | 3.199 | -10.72 | 3.45 | 1.793 | K point | K point |
| XZ | 3.205 | -13.3 | 3.05 | 2.052 | $\Gamma-K$ route | K point |
| XZ′ | 3.198 | -3.77 | 3.68 | 1.946 | K point | K point |

**References:**

1. Xu, Z., Li, Y., Li, C. & Liu, Z. Tunable electronic and optical behaviors of two-dimensional germanium carbide. *Appl. Surf. Sci.* **367**, 19–25 (2016).

2. Ji, Y., Dong, H., Hou, T. & Li, Y. Monolayer graphitic germanium carbide (g-GeC): The promising cathode catalyst for fuel cell and lithium-oxygen battery applications. *J. Mater. Chem. A* **6**, 2212–2218 (2018).

3. Wu, X., Zhang, W., Yan, L. & Luo, R. The deposition and optical properties of Ge1-xCx thin film and infrared multilayer antireflection coatings. *Thin Solid Films* **516**, 3189–3195 (2008).

4. Yang, J. *et al.* High-Performance p-type 2D FET Based on Monolayer GeC with High Hole Mobility: A DFT-NEGF Study. **8**, 2200388(1–8) (2022).

5. Fan, X., Jiang, J., Li, R. & Mi, W. Half-metallicity and magnetic anisotropy in transition-metal-atom- doped graphitic germanium carbide (g-Gec) monolayers. *J. Phys. Chem. C* (2021) doi:10.1021/acs.jpcc.1c04139.

6. Şahin, H. *et al.* Monolayer honeycomb structures of group-IV elements and III-V binary compounds: First-principles calculations. *Phys. Rev. B - Condens. Matter Mater. Phys.* **80**, (2009).

7. Wang, S. *et al.* First-principles calculations of aluminium nitride monolayer with chemical functionalization. *Appl. Surf. Sci.* **481**, 1549–1553 (2019).

8. Zamil, M. Y., Islam, M. S., Stampfl, C. & Park, J. Tribo-Piezoelectricity in Group III Nitride Bilayers: A Density Functional Theory Investigation. *ACS Appl. Mater. Interfaces* (2022) doi:10.1021/acsami.2c00855.
